# Transforming Dental Care Through Empathetic and Clear Communication: A Comprehensive Review and Implementation Framework

**DOI:** 10.3390/dj14020111

**Published:** 2026-02-13

**Authors:** Jasmine Cheuk Ying Ho, Joanna Cheuk Yan Hui, Hollis Haotian Chai, Michelle Zeping Huang, Edward Chin Man Lo, Chun Hung Chu

**Affiliations:** 1 Faculty of Dentistry, University of Hong Kong, Hong Kong 852, Chinamichellehuang@hsu.edu.hk (M.Z.H.);; 2 Department of English, The Hang Seng University of Hong Kong, Hong Kong 852, China

**Keywords:** dentist, patient, communication, digital, empathy, satisfaction, modern, patient-centred, quality of care

## Abstract

Effective dentist-patient communication underpins care, empowering informed decisions, reducing anxiety, improving efficiency, and fostering trust through clear, accurate, cohesive exchanges. This narrative review used a structured Medline search of literature, employing key terms to select and synthesize relevant English-language publications on dentist-patient communication without strict inclusion criteria. Key strategies include active listening, empathetic dialogue, patient-centred approaches, and the use of plain language and visual aids to demystify complex information. Additionally, integrating technology for appointment reminders, virtual consultations, and feedback mechanisms can streamline interactions. Crucially, cultural competency and sensitivity to individual needs ensure inclusivity and personalized care. Building on these findings, the study outlines ten actionable pillars for effective communication: (1) Initial Consultation: Establish rapport and gather comprehensive medical/dental histories. (2) Treatment Explanation: Simplify diagnoses and options using layman’s terms. (3) Informed Consent: Transparently discuss risks/benefits and invite questions. (4) Patient Education: Clarify oral hygiene practices and post-treatment expectations. (5) Anxiety Management: Address fears through reassurance and tailored coping strategies. (6) Follow-Up Care: Maintain post-treatment engagement to resolve concerns. (7) Feedback Systems: Leverage patient insights for service improvement. (8) Cultural Sensitivity: Adapt communication to diverse backgrounds. (9) Non-Verbal Cues: Employ positive body language and active listening. (10) Technology Integration: Utilize digital tools for efficiency and accessibility. By prioritizing empathy, clarity, and adaptability, clinicians can transform dental visits from anxiety-inducing encounters into collaborative partnerships. This approach not only elevates patient satisfaction and adherence but also redefines the standard of care, aligning clinical practice with the evolving needs of modern dentistry.

## 1. Introduction

Effective communication between dentists and patients is fundamental to providing high-quality dental care [1]. It forms the foundation of trust, enabling patients to feel comfortable, understood, and confident in their treatment plans. Clear dialogue ensures that patients fully comprehend their oral health conditions, the procedures involved, and the importance of follow-up care, which can significantly improve treatment adherence and outcomes [2]. Good communication also allows dentists to better assess patients’ concerns, anxieties, and preferences, fostering a patient-centred approach. When patients feel heard and respected, they are more likely to share vital information about their symptoms, habits, and medical histories, which is essential for accurate diagnosis and personalized treatment [3]. Moreover, effective communication can reduce dental anxiety, a common barrier to seeking care, by providing reassurance and addressing fears proactively [1,4].

The rapid development of digital communication tools, such as emails, text messaging, practice websites, and secure patient portals, has transformed dentist–patient communication. These platforms allow patients to receive appointment reminders, pre- and post-operative instructions, and personalized education materials at convenient times. Video consultations and messaging systems also enable timely clarification of concerns, reducing misunderstandings and anxiety. By supporting continuous, accessible, and documented dialogue, digital communication strengthens engagement, builds trust, and promotes shared decision-making in oral health care for better outcomes.

Strong dentist-patient communication enhances overall satisfaction, encourages ongoing engagement, and builds long-term relationships [5]. It bridges cultural, linguistic, and emotional gaps, ensuring that diverse patient populations receive inclusive, respectful, and empathetic care. Ultimately, effective communication is not just a skill but a critical component of ethical, compassionate, and successful dental practice [6]. As in all healthcare disciplines, communication is the cornerstone of quality care, and dentistry is no exception [7].

A dentist’s ability to convey information clearly and compassionately can mean the difference between a patient who feels empowered and one who leaves the clinic. Yet, dental visits are often fraught with anxiety, misunderstandings, and unmet expectations [8]. The aims of this study are to study the critical role of effective dentist-patient communication, synthesizing evidence-based strategies into essential topics that clinicians can adopt to enhance trust, reduce fear, and improve clinical outcomes.

## 2. Method

This narrative review followed a structured approach to find and synthesize literature on dentist-patient communication. A comprehensive search was carried out using Medline, a main database for dental and medical research. The search covered English-language articles available up to 31 December 2025. Keywords from initial sources were used to refine the strategy. Frequently used terms included “communication,” “empathy,” “dental,” “dentist,” and “patient.” Publications were chosen for their relevance and contribution to the synthesis rather than strict inclusion criteria. The selected articles were summarized and combined to provide a thorough overview of dentist-patient communication.

The authors conducted a structured Medline search and selected relevant articles on dentist and patient communication. They summarized each study and identified recurring themes, such as rapport building, clear explanations, consent, education, anxiety management, follow up, feedback, cultural sensitivity, nonverbal skills, and technology use. They then grouped these themes into ten practical pillars that reflected the most consistently supported strategies.

Narrative reviews present an integrated summary of the literature instead of separated or strictly systematic results. Therefore, the findings and discussion are combined throughout the text to provide context, interpretation, and critical analysis. This format offers a coherent view of current knowledge and emerging trends in the field. Relevant studies are cited throughout the manuscript. Because this is a narrative review, no formal documentation of the search process was required. References are provided for all included studies.

## 3. The Foundation of Effective Communication

**Figure 1:**
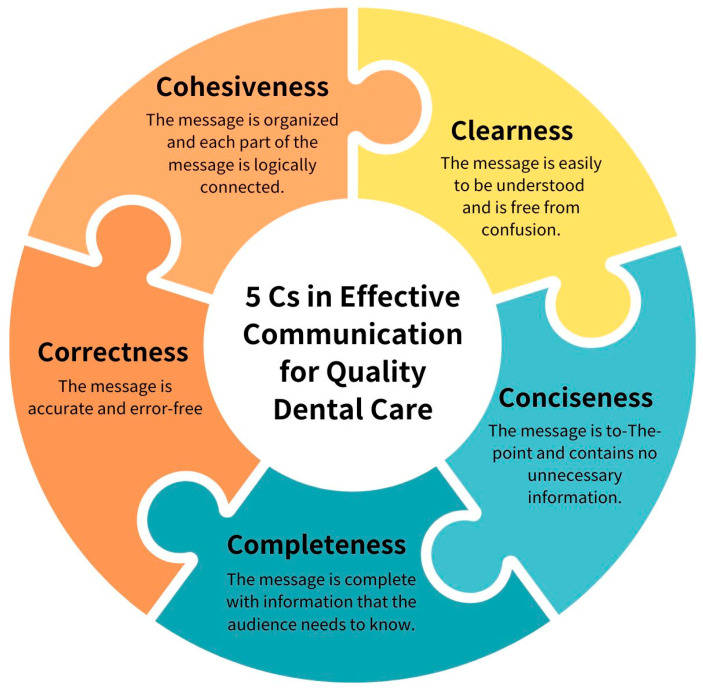
The 5 C’s framework for effective dentist–patient communication: clarity, correctness, conciseness, completeness, and cohesiveness [9].

Effective dentist–patient communication is a dynamic, bidirectional process guided by five fundamental principles—clarity, accuracy, conciseness, completeness, and cohesion—collectively known as the “5 C’s” of communication [9,10] (Figure 1).

These principles ensure that interactions are not merely transactional but transformative. When implemented effectively, quality communication empowers patients to make informed decisions about their oral health, increases adherence to treatment plans, and builds lasting trust [10]. Studies underscore that effective communication correlates with higher patient satisfaction, reduced no-show rates, and even better clinical outcomes, such as improved oral hygiene practices [11]. Conversely, communication breakdowns can trigger a cascade of adverse consequences, including patient misunderstandings, heightened anxiety, and decreased satisfaction with care. These communication breakdowns discourage patients from seeking timely dental treatment and contribute to preventable medical errors, including misdiagnoses, medication administration errors, and treatment delays [12].

## 4. Beyond Teeth: Understanding the Person

Considering cultural and social factors during the initial consultation allows dentists to build trust quickly, personalize information, and uncover health beliefs that shape expectations [13]. When dentists ask open questions about language preferences, family roles, or past dental experiences, they signal respect and gather context that guides treatment explanations. Vigilance against prejudice and judgement must become part of daily practice; clinicians should reflect on their assumptions, notice implicit reactions, and adjust their communication to avoid dismissing concerns rooted in culture or socioeconomic stressors. This attentive stance reduces misinterpretation, improves consent discussions, and encourages patients to share sensitive details—such as traditional remedies or financial constraints—ultimately aligning care plans with each patient’s reality.

Dentists should outline estimated costs at the outset so patients understand the financial scope of treatment. Clear price discussions prevent surprise bills and demonstrate respect for patients’ resources [14]. Clinicians must describe all viable treatment possibilities, including benefits and limitations, to support shared decision-making [15]. Dentists should invite questions about lifestyle, work schedules, and support systems to gauge the level of engagement required. Before patients decide, clinicians should arrange follow-up to revisit concerns, reinforce instructions, and confirm readiness for care. Transparent timelines help patients plan time off, finances, and family responsibilities. Documenting agreements in plain language strengthens accountability and reassures patients that their choices are central to the process.

Understanding patients’ social, cultural, and economic vulnerabilities is essential for delivering ethical, realistic, and patient-centred dental care [16]. Awareness of cultural norms and beliefs helps dentists explain diagnoses and treatments in ways that respect patients’ values and avoid misunderstandings. Recognizing economic constraints allows clinicians to propose phased, affordable, and prioritized treatment options rather than ideal but unattainable plans. Sensitivity to social stressors—such as unstable housing, caregiving responsibilities, or limited transport—guides appropriate scheduling, follow-up, and preventive strategies. This holistic understanding builds trust, improves adherence, and reduces the risk that patients feel judged, excluded, or unable to access necessary dental treatment.

## 5. Challenges in Dental Communication

Effective communication lies at the heart of quality dental care; dental practice settings often present complex interpersonal and systemic barriers [17]. Patients may enter the clinic with pre-existing anxieties, including fears of pain or concerns about potential judgement regarding their oral health status. Additionally, the technical complexity of dental jargon can alienate those unfamiliar with terms [18,19].

**Figure 2:**
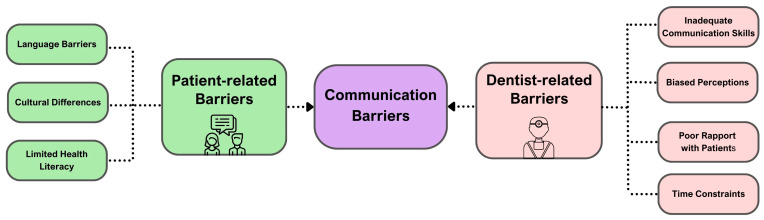
Key barriers to dentist–patient communication.

Figure 2 illustrates the communication challenges in dentistry emerge from multiple, interlinked domains: rapport failure, time constraints, health literacy, dentists’ skills and perceptions, language barriers, and cultural differences. Time constraints in busy practices restrict opportunities to build relationships or explain treatment options thoroughly, leading to superficial conversations that leave patients uncertain or anxious [19]. Rapport failure, often rooted in limited trust or empathy, further distances patients from clinicians [20,21].

Health literacy barriers compound the patients’ struggle to comprehend oral-health information, postoperative instructions, or preventive guidance, reducing adherence to care recommendations [22]. From the clinician’s perspective, inadequate communication skills or perception biases can negatively affect interactions, particularly when practitioners misinterpret patients’ emotional cues or preferences.

Meanwhile, cultural and language differences may hinder mutual understanding; beliefs about oral health, esthetics, and decision-making authority vary greatly across communities [22]. For example, some older or immigrant patients may prioritize functional comfort over cosmetic appearance or prefer that family members participate directly in treatment discussions. Figure 2 summarizes the key barriers to effective dentist–patient communication [1]. Collectively, these factors underscore the need for adaptable, person-centred communication strategies.

## 6. A Pathway to Improvement in Communication

A review of existing literature reveals a consensus on key strategies for overcoming these challenges; active listening, empathy, and plain language emerge as priorities [22,23]. Visual aids, such as intraoral cameras or digital models, bridge gaps in understanding by making abstract concepts tangible [24]. Meanwhile, cultural competency training and patient-centred approaches ensure care is tailored to individual needs. Technology also plays a growing role, with tools like appointment reminders and telehealth consultations enhancing accessibility [25].

Underpinning all these strategies is the principle of *continuity*, the communication must extend beyond the clinic chair to follow-ups and feedback loops that sustain patient engagement. This paper distils these evidence-based strategies into ten actionable topics for clinicians, each addressing a specific facet of the dentist-patient relationship. Table 1 summarizes ten actionable topics for effective dentist-patient communication.

The ten pillars of effective dentist–patient communication form a framework for safe, ethical, and collaborative care. First, respect and empathy acknowledge the patient as a person, not just a case. Active listening allows dentists to understand fears, expectations, and priorities. Clear, jargon-free language ensures patients grasp diagnoses, options, and instructions. Cultural and social sensitivity helps tailor explanations and avoid prejudice or misunderstanding. Shared decision-making invites patients into treatment choices, reinforcing autonomy. Transparency about benefits, risks, costs, and alternatives builds trust and prevents conflict. Professionalism and confidentiality create a safe space for honest disclosure. Support for patient engagement—encouraging questions, checking understanding, and motivating self-care—improves adherence. Attention to nonverbal cues, such as eye contact and a calm tone, reduces anxiety and builds rapport. Finally, continuity and follow-up—summarizing plans, confirming next steps, and checking outcomes—strengthen long-term relationships and improve oral health results. Together, these pillars promote communication that is ethical, patient-centred, and clinically effective.

### 6.1. Initial Consultation: Building the Foundation of Trust and Collaboration

The initial consultation establishes the cornerstone of the dentist-patient relationship, shaping perceptions and expectations for subsequent care [26]. Beyond clinical assessment, this phase emphasizes psychological and emotional engagement to mitigate apprehension often associated with dental settings. The clinic’s environment, characterized by calming esthetics and courteous staff, often plays a pivotal role in reducing patient anxiety, while clinicians’ approachability, conveyed through attentive body language and culturally sensitive greetings, fosters immediate rapport. Research underscores that patients who perceive respect and empathy during this stage exhibit higher compliance rates and long-term loyalty, highlighting the correlation between trust-building and clinical outcomes [27].

Central to this process is the medical and dental history intake, which should transcend routine documentation to become an opportunity for collaboration. Active listening through verbal affirmations and reflective responses enables clinicians to decode not only symptoms but also unspoken concerns, such as financial constraints or procedural fears. By prioritizing open-ended inquiry, dentists invite patients to articulate their priorities, lifestyle influences, and health contexts, ensuring care plans align with individual needs [28]. This collaborative approach reinforces the patient’s agency, positioning them as an active contributor rather than a passive recipient.

The consultation culminates in shared decision-making, where clarity and transparency bridge the gap between clinical expertise and patient understanding [29]. Summarizing findings in accessible language demystifies diagnoses and treatment pathways, while inviting questions empowers patients to voice uncertainties [4,30]. This exchange cultivates mutual respect, balancing technical precision with emotional support [31]. Ultimately, a well-executed initial consultation transcends transactional care, laying the groundwork for enduring partnerships that prioritize both oral health and patient dignity [30]. By harmonizing human connection with professional rigour, clinicians transform apprehension into advocacy, ensuring patients depart not only informed but also invested in their care journey.

### 6.2. Treatment Explanation: Bridging Knowledge Gaps for Empowered Decision-Making

Effective treatment explanations translate technical dental concepts into patient-centred dialogue, fostering collaboration and informed consent [32]. The use of specialized terminology often creates barriers, leaving patients feeling excluded from their own care. Clinicians should prioritize plain language, reframing diagnoses and procedures through universally relatable concepts [10]. Analogies grounded in everyday experiences enhance comprehension, enabling patients to visualize conditions and their implications without requiring prior medical knowledge. This approach not only clarifies the rationale and urgency of treatment but also ascribes tangible meaning to abstract diagnoses. Studies highlight the importance of clear, iterative communication, as patients often struggle to retain critical clinical information post-consultation [33].

Visual aids complement verbal explanations by offering multisensory engagement [34]. Intraoral imaging, 3D models, and dynamic digital tools transform invisible or complex issues, such as decay progression or orthodontic outcomes into accessible visuals [35,36]. These resources cater to diverse learning preferences, allowing patients to observe anatomical relationships and treatment mechanics [37]. By contextualizing interventions within personal health goals, visual demonstrations align clinical objectives with patient priorities, whether functional restoration or esthetic improvement.

Active verification of understanding ensures alignment between clinician intent and patient interpretation. The “teach-back” method, which patients restate information in their own words, identifies knowledge deficits while reinforcing critical details [38]. Inviting open-ended questions further dismantles barriers, creating space for discussions about risks, alternatives, and logistical concerns. Patients who fully comprehend their care plan demonstrate higher adherence to postoperative protocols and preventive practices, reducing complications and fostering trust [39]. Ultimately, transparent communication transforms treatment explanations from obligatory disclosures into collaborative dialogues [40]. By demystifying dentistry’s complexities, clinicians empower patients to make decisions rooted in clarity rather than uncertainty, strengthening therapeutic alliances and improving long-term health outcomes.

### 6.3. Informed Consent: Elevating Ethical Practice Through Collaborative Dialogue

Informed consent transcends administrative formalities, serving as an ethical imperative that centres patient autonomy in clinical decision-making [15]. This process requires clinicians to clearly articulate treatment risks, benefits, alternatives, and potential outcomes of non-intervention, ensuring patients grasp the implications of their choices [1]. By contextualizing medical complexities into accessible terms, dentists empower individuals to align care with personal priorities, whether in financial, functional, or quality-of-life considerations.

Effective consent dialogues require a balance of precision and empathy [41]. Clinicians should avoid overwhelming technical details, instead tailoring explanations to address patients’ unique concerns and lifestyles. Open-ended inquiries uncover unspoken fears or logistical barriers, while the teach-back method—asking patients to summarize key points—verifies understanding and corrects misinterpretations [42]. Cultural competence is paramount: for populations hesitant to question authority, proactive reassurance encourages dialogue, ensuring consent reflects genuine comprehension rather than passive acquiescence.

Robust consent practices yield measurable benefits. Research indicates that patients who receive thorough explanations report significantly higher satisfaction and are less likely to pursue litigation, even when complications occur [43,44]. Vulnerable groups, including those with limited health literacy or cognitive challenges, particularly benefit from this collaborative approach, as it mitigates power imbalances and fosters advocacy [45]. By reframing consent as an ongoing conversation rather than a transactional checkbox, clinicians strengthen trust and reinforce ethical practice. This patient-driven model not only fulfils legal obligations but also cultivates therapeutic alliances rooted in transparency and mutual respect. Ultimately, informed consent becomes a dynamic tool for honouring patient agency, transforming regulatory compliance into a catalyst for equitable, dignified care [46].

### 6.4. Patient Education: Empowering Lifelong Oral Health Through Knowledge

Patient education transforms reactive care into proactive prevention, addressing the reality that over half of dental diseases are avoidable through informed self-care [47,48]. By equipping patients with personalized, evidence-based strategies, clinicians empower individuals to disrupt cycles of neglect and reduce recurrence of issues like caries and periodontitis [49]. Tailored guidance accounts for medical histories, lifestyles, and socioeconomic factors—ensuring recommendations align with patients’ capacities and constraints [50]. This preventive focus not only minimizes future complications but also reduces long-term financial burdens, fostering autonomy and confidence in managing oral health.

Effective education prioritizes engagement over instruction. Interactive tools such as anatomical models, digital demonstrations, or habit-tracking apps translate abstract concepts into actionable routines [51]. Clinicians must move beyond generic advice and instead demonstrate techniques and clarify misconceptions through visual or hands-on learning. Post-treatment reinforcement is equally vital, with structured guidelines addressing activity restrictions and warning signs to monitor [52]. The teach-back method verifies comprehension, while adaptable formats ensure accessibility across diverse needs.

Cultural competence amplifies the impact of education. Dietary counselling should acknowledge cultural food practices while addressing nutritional risks relevant to oral health. Similarly, clinicians must navigate traditional practices with sensitivity, offering science-based alternatives without dismissing cultural values. By framing education as collaborative problem-solving—not prescriptive mandates—dentists address barriers like time limitations or resource access that hinder adherence. Investing in patient education yields measurable dividends: studies indicate educated patients exhibit higher adherence to hygiene protocols and fewer urgent interventions [53]. Ultimately, this approach redefines oral health as a partnership, equipping individuals with the knowledge to convert daily choices into enduring wellness [54].

### 6.5. Addressing Concerns and Anxiety: Building Trust Through Empathetic, Tailored Care

Dental anxiety impacts 60% of adults globally, with 15% avoiding care due to fear [55,56]. Dental anxiety often leads to severe oral and systemic health consequences [57]. This fear frequently stems from past trauma, perceived loss of control, or sensory triggers. Acknowledging these emotions openly (“Your feelings are valid; let us adjust our approach to help you feel safe”) builds trust and shifts dynamics from paternalistic to collaborative.

Clinics can foster safety through environmental and procedural adaptations. Reducing sensory overload by calming music, adjustable lighting, or non-clinical scents to create a less intimidating atmosphere [58]. Grounding tools like weighted blankets offer tactile comfort, while clear, step-by-step explanations demystify treatments. For heightened anxiety, gradual exposure through non-treatment visits or controlled breathing exercises helps patients acclimate [59]. Sedation options, presented without judgement, provide additional support for those needing extra relaxation.

Staff training ensures consistency in compassionate care. Frontline teams should use reassuring language, avoiding dismissive phrases like “This won’t hurt” in favour of honest, empowering communication (“You’ll feel pressure, but we’ll stop anytime you signal”) [60]. Visual or verbal cue systems allow patients to control the pace of care without speaking. Post-appointment encouragement reinforces positive experiences, fostering resilience over time [61]. Research shows such strategies reduce anxiety and demonstrates that emotional safety is as vital as clinical expertise [62]. By prioritizing psychological comfort alongside technical precision, dental professionals transform fear into empowerment, ensuring patients feel respected, heard, and capable of pursuing lifelong oral health.

### 6.6. Follow-Up Communication: Sustaining Care Beyond the Chair

Follow-up communication transforms isolated dental treatments into enduring care partnerships, addressing the critical post-procedural period when approximately one-third of complications arise [63]. Many patients delay reporting issues due to uncertainty or fear of inconvenience, risking preventable setbacks. Proactive check-ins—whether calls, texts, or portal messages—demonstrate commitment to patient well-being while enabling early intervention [64]. Personalized updates outlining recovery milestones and warning signs empower individuals to navigate healing confidently, reducing avoidable emergencies [65].

Effective systems balance automation with human touch. Templated messages can efficiently track symptoms through simple response prompts (e.g., pain level scales), while high-risk patients benefit from direct clinician contact to address complex needs. Secure digital platforms allow photo-based progress assessments, minimizing in-person visits. Clear, jargon-free language ensures accessibility: asking about “bleeding” rather than “haemorrhage” prevents confusion and encourages honest feedback [37,64]

Consistent follow-ups yield both clinical and relational rewards [65]. Research demonstrates that patients value post-visit contact as an important indicator of compassionate and comprehensive care [66]. However, respect for patient autonomy is paramount—offering opt-out options and tailoring frequency to individual preferences prevents communication fatigue. Integrating follow-ups into electronic health records streamlines workflows, ensuring no case slips through administrative cracks [65]. By prioritizing timely, empathetic engagement, clinics reinforce trust and accountability. This approach not only mitigates health risks but also positions patients as active collaborators in their long-term wellness, fostering loyalty that extends far beyond a single appointment.

### 6.7. Feedback Mechanisms: Fostering Growth Through Patient-Centered Dialogue

Feedback systems empower dental practices to evolve by centering patient experiences as catalysts for improvement. In a field where trust defines success, actively seeking critiques—via surveys, conversations, or digital platforms—signals respect for patient perspectives and a commitment to addressing systemic gaps [65]. Proactive solicitation of input reveals overlooked issues, from logistical frustrations to communication breakdowns, while dismantling the hierarchical dynamics that often marginalize patient voices [67]. Digital real-time feedback systems show promising patient engagement, with studies indicating that patients appreciate the immediacy of feedback compared to traditional surveys, helping to offset ‘feedback fatigue’ while enabling healthcare organizations to respond promptly to patient concerns [68].

Effective feedback strategies prioritize accessibility and responsiveness. Digital tools can analyze trends in patient responses, identifying urgent concerns such as recurring anxiety or confusion [68]. In-person check-ins during or after appointments capture immediate reactions with emotional nuance. Inclusive formats, including visual rating systems and multilingual options, ensure diverse populations can contribute comfortably. The true measure of success lies in applying this data: revising protocols based on common critiques and publicly sharing updates to demonstrate accountability. Transparent communication about changes reinforces that patient input drives tangible progress [69].

Addressing feedback challenges requires creativity and empathy. Incentives boost participation rates without compromising authenticity, while staff training in non-defensive response techniques transforms criticism into learning opportunities. Integrating feedback with broader practice initiatives—such as refining pain management guides or anxiety-reduction strategies—ensures systemic alignment. By embedding feedback into daily operations, clinics foster a culture where continuous improvement becomes inseparable from patient care, turning every perspective into a building block for excellence [70].

### 6.8. Cultural Competency: Building Trust Through Inclusive, Respectful Care

Cultural competency in dental care involves more than tolerance; it requires a proactive understanding of how patients’ beliefs, traditions, and social contexts influence their health behaviours [64]. Studies indicate that over 20% of patients from non-Western backgrounds avoid dental visits due to fears of cultural insensitivity, such as dismissive attitudes toward family involvement or language barriers [71]. For example, in many communities, healthcare decisions are collective, and ignoring this norm by addressing only the individual patient can undermine trust. Additionally, some Indigenous cultures perceive oral health as interconnected with spiritual well-being, necessitating holistic communication approaches [72,73].

Effective strategies include employing trained interpreters instead of family members during sensitive discussions, providing multilingual educational materials, and adjusting clinical practices to accommodate cultural norms—such as scheduling around fasting periods or recognizing cultural preferences regarding treatments [72]. Technological tools like multilingual intake forms and culturally tailored video content further enhance understanding and comfort.

Addressing cultural nuances requires comprehensive staff training in cultural competency, including recognition of non-verbal communication patterns, understanding diverse religious and spiritual beliefs, and acknowledging how cultural backgrounds influence health-seeking behaviours and treatment adherence [74]. Collaborations with cultural liaisons and local organizations support ongoing learning and sensitivity. Incorporating cultural considerations into all aspects of care—such as respecting herbal remedies, accommodating family involvement, and selecting appropriate feedback methods—fosters trust and inclusivity. By embedding cultural awareness into every interaction, dental practices can transform into welcoming spaces that honour diverse identities, thereby improving patient engagement, adherence, and overall health outcomes [75].

### 6.9. Non-Verbal Cues: The Silent Language of Trust in Dental Care

Non-verbal communication plays a vital role in establishing trust and reducing anxiety in dental care. Non-verbal communication plays a vital role in establishing trust and reducing anxiety in dental care. Research indicates that most patients perceive practitioners with open body language, such as uncrossed arms and a relaxed posture, as more competent, regardless of technical skill [76]. From the moment patients enter the clinic, welcoming gestures like a warm smile and appropriate eye contact can help lower stress levels [77]. During treatment, controlled movements, deliberate pauses for eye contact, and visible facial expressions—especially when masks obscure smiles—signal attentiveness and reassurance [77]. Gentle touch, when culturally appropriate, can further establish a human connection, alleviating anxiety.

Mastering non-verbal cues requires intentionality and adaptability. For pediatric patients, aligning posture to eye level and using expressive facial gestures fosters rapport [78]. For older adults, slow, deliberate movements and affirming nods enhance understanding [79]. Mirroring a patient’s breathing or subtle gestures can subconsciously communicate empathy [80]. However, distractions such as checking the clock or appearing impatient through body language can undermine trust. To adapt to mask-wearing, some practices use transparent shields or emphasize vocal warmth—melodic tones and pauses—to compensate for hidden facial expressions.

Cultural sensitivity influences non-verbal interactions; for example, avoiding prolonged eye contact with hierarchical cultures or being mindful of gestures with cultural taboos. During anxiety management, synchronized breathing and calm hand signals effectively de-escalate distress. Even in follow-up interactions, brief pauses before responding demonstrate attentiveness. By refining non-verbal communication, dental teams can create a reassuring environment where every gesture and posture conveys safety, empathy, and professionalism, enhancing the overall patient experience [81].

### 6.10. Technological Integration: Revolutionizing Dental Dialogue in the Digital Age

Technological integration is transforming dental communication and care delivery in the digital age. With approximately 60% of patients preferring appointment booking via apps over phone calls, digital tools facilitate more efficient and accessible interactions [82]. AI-powered chatbots handle after-hours inquiries, providing immediate responses to common questions, while virtual consultations enable patients and caregivers to discuss treatment progress remotely, increasing convenience [83]. Patient portals serve as comprehensive platforms for viewing 3D scans, making payments, and accessing educational content, thereby streamlining administrative tasks and reducing front-desk workload.

Beyond efficiency, technology fosters transparency and trust. Real-time updates during procedures keep patients and their families informed, alleviating anxiety. Visualization tools, such as augmented reality overlays, help patients understand how proposed treatments will impact their appearance, enhancing engagement and decision-making [84]. However, challenges include interface accessibility for older adults and maintaining data security. Hybrid options like tablet kiosks with simplified interfaces and encrypted platforms ensure inclusivity and compliance with privacy standards.

Integrating technology across various care pillars amplifies its benefits. Digital surveys embedded in apps increase response rates, providing valuable feedback to improve service quality [85]. AI analysis of patient feedback identifies common concerns, guiding targeted improvements. Pre-visit virtual reality modules help patients with dental phobias acclimate to procedures [86]. Importantly, technology serves to augment the human connection—when practitioners review app-submitted data or use tele-dentistry to reassure vulnerable patients, it enhances personalized care [87]. Ultimately, digital tools act as silent partners that expand the reach, transparency, and effectiveness of dental care without replacing the essential human touch.

## 7. The Path Forward

These ten topics are not standalone tactics but interconnected components of a holistic communication framework. By adopting them, clinicians can transform routine appointments into opportunities for education, collaboration, and trust-building. In an era where patient-centred care is paramount, refining communication skills is not just beneficial—it is essential for the future of dental practice [88]. Ultimately, the goal is to shift the narrative of dental visits from dread to confidence, from confusion to clarity, and from transactional interactions to partnerships rooted in mutual respect.

## 8. Conclusions

Effective communication is not merely a soft skill: it is the heartbeat of exceptional dental care. Dental schools should embed longitudinal communication training that blends simulation, cultural responsiveness, and digital engagement skills so graduates can operationalize these pillars from their first patient encounters. By prioritizing empathy, clarity, and adaptability, clinicians can transform dental visits from anxiety-inducing encounters into collaborative partnerships. This approach not only elevates patient satisfaction and adherence but also redefines the standard of care, aligning clinical practice with the evolving needs of modern dentistry.

## Figures and Tables

**Table 1:** Ten Actionable Topics for Effective Dentist-patient Communication.

1. **Initial Consultation** - Establishing rapport and a welcoming environment. - Gathering comprehensive medical and dental histories. 2. **Treatment Explanation** - Clearly explain diagnoses and treatment options. - Using layman’s terms to ensure understanding. 3. **Informed Consent** - Discussing potential risks and benefits. - Encouraging patients to ask clarifying questions. 4. **Patient Education** - Providing information on oral hygiene practices. - Explaining post-treatment care and expected outcomes. 5. **Addressing Concerns and Anxiety** - Recognizing common dental anxieties and fears. - Offering methods to reduce anxiety (e.g., sedation options, calming techniques). 6. **Follow-Up Communication** - Checking in post-treatment to address any concerns. - Encouraging ongoing communication regarding dental health. 7. **Feedback Mechanism** - Encouraging patients to provide feedback on their experience. - Using feedback for service improvement. 8. **Cultural Competency** - Being aware of and sensitive to cultural differences. - Tailoring communication approaches to diverse patient backgrounds. 9. **Non-Verbal Communication** - Maintaining appropriate body language and eye contact. - Using active listening to reinforce understanding and empathy. 10. **Technological Integration** - Utilizing digital communication tools for appointment reminders and follow-ups. - Offering virtual consultations when appropriate.

## Data Availability

No new data were created or analyzed in this study.
